# Bedside transcranial sonography monitoring in a patient with hydrocephalus post subarachnoid hemorrhage

**DOI:** 10.1186/s13089-017-0072-1

**Published:** 2017-09-27

**Authors:** Ahmed Najjar, André Y. Denault, Michel W. Bojanowski

**Affiliations:** 1 Department of Neurosurgery, Centre Hospitalier de l’Université de Montréal, Montreal, QC Canada; 2 Division of Critical Care, Centre Hospitalier de l’Université de Montréal, Montreal, QC Canada; 3 Department of Anesthesiology, Montreal Heart Institute, Université de Montréal, 5000 Belanger Street, Montreal, QC H1T 1C8 Canada

**Keywords:** Transcranial ultrasound, Subarachnoid hemorrhage, Intracranial hypertension, Bedside ultrasound, Transcranial sonography

## Abstract

**Background:**

Development of hydrocephalus can occur after subarachnoid hemorrhage (SAH). Typically, it is diagnosed with computed tomography, CT, scan. However, transcranial sonography (TCS) can be used particularly in patients with craniotomy which removes the acoustic interference of the skull and allows a closer up visualization of brain structures through the skin.

**Case presentation:**

We report a 73-year-old woman who was hospitalized for SAH and developed acute hydrocephalus requiring an external ventricular drain (EVD). In this patient, detection and monitoring of hydrocephalus was done and monitored with a small pocket-sized TCS device. Nine days after surgery, weaning of the EVD was attempted. Prior to EVD closure and removal, TCS showed a measurement of the 3rd ventricle at around 1.16 cm. On the third day, the patient deteriorated clinically and the TCS showed a dilated 3rd ventricle measuring 1.37 cm which correlated well with computed tomography and with clinical signs of active hydrocephalus as both her sensorium and communication were affected. Subsequently following EVD re-installation, on the next day, TCS showed that the 3rd ventricle dimension was reduced to 0.99 cm and the following day it went down to 0.69 cm.

**Conclusions:**

Patients with SAH and in particular those with a craniotomy can be monitored easily at the bedside with hand-held TCS for the development and monitoring of hydrocephalus.

## Background

Hydrocephalus in patients with subarachnoid hemorrhage (SAH) is a frequent cause of clinical deterioration. In addition to clinical evaluation, computed tomography (CT) scans are used regularly to monitor for the appearance of hydrocephalus. The use of bedside ultrasound for imaging has expanded to multiple clinical applications especially in the acute critical patient care. We describe a case for which we used a hand-held two-dimensional (2D) transcranial sonography (TCS) probe to follow-up a patient with hydrocephalus after subarachnoid hemorrhage demonstrating high accuracy and clinical correlation. Permission to use the following figures was granted by the patient’s family.

## Case report

**Fig. 1:**
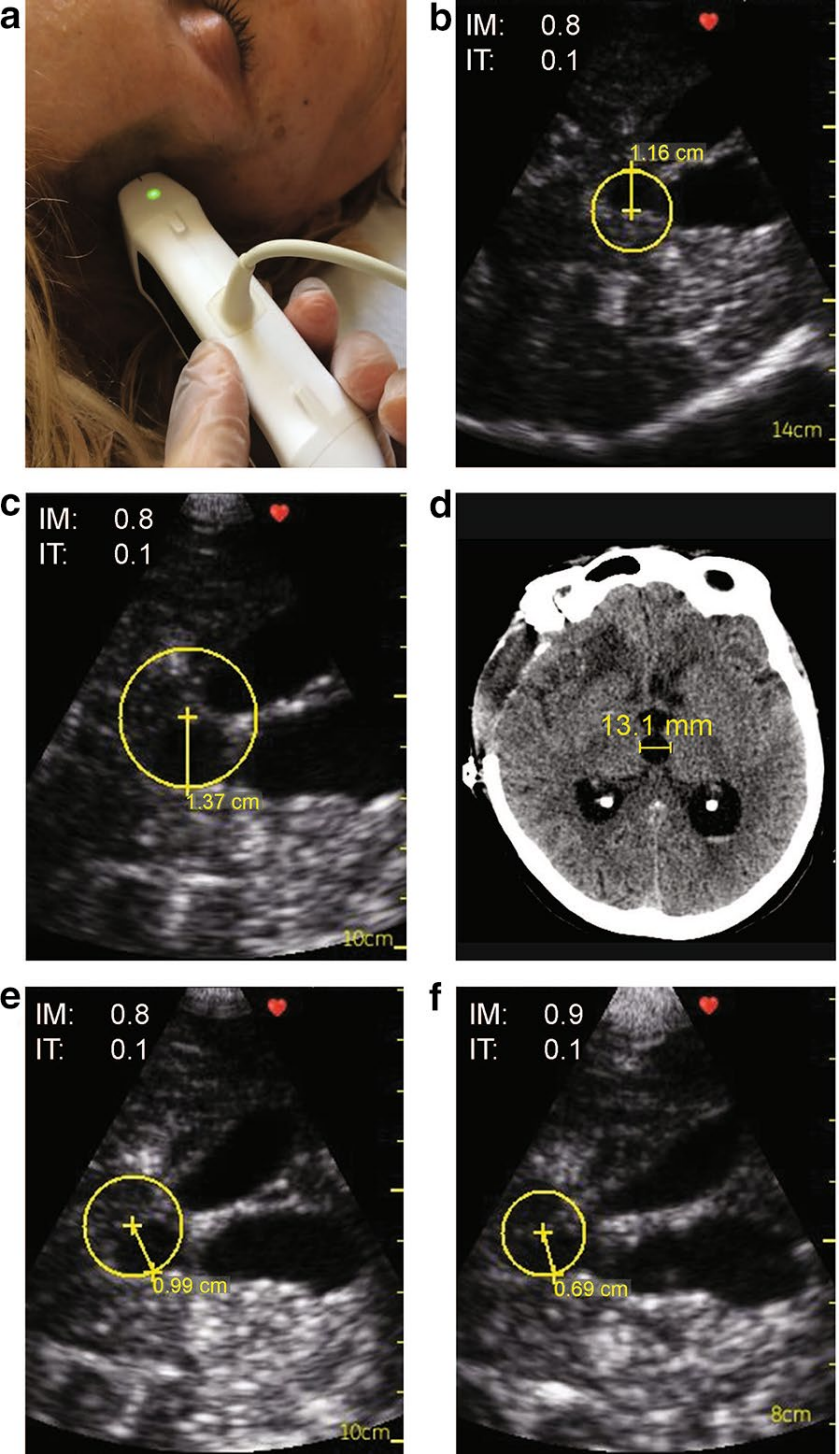
**a** TCS using hand-held pocket ultrasound device (GE Vingmed Ultrasound AS, Horten, Norway). **b** Prior to EVD closure, TCS showed a measurement of the 3rd ventricle at around 1.16 cm. **c** On the third day, TCS showed a dilated 3rd ventricle measuring 1.37 cm. **d** CT scan showed a dilated 3rd ventricle measuring 13.1 mm. **e** Subsequently following EVD re-opening, on the next day using TCS the 3rd ventricle dimension was reduced to 0.99 cm. **f** The following day it went down to 0.69 cm

A 73-year-old woman was admitted to our hospital in coma after having an acute thunder clap headache. Her vitals were stable apart from high blood pressure and other signs of increased intracranial pressure (ICP). Her CT showed extensive SAH with acute hydrocephalus and signs of elevated ICP. An anterior communicating artery aneurysm was identified on CT angiogram. Patient underwent urgent surgery including insertion of external ventricular drain (EVD), right-sided craniectomy and clipping of the aneurysm at the same time. She had a fair good outcome after the surgery immediately with improvement in consciousness and good control of her ICP. An intensivist with bedside ultrasound experience followed the patient with daily TCS using a hand-held pocket ultrasound device (GE Vingmed Ultrasound AS, Horten, Norway) (Fig. 1a). Image acquisition was facilitated by the absence of bone on the site of the craniectomy which allows close contact with the brain structure through the skin. The technique is usually straight forward. In contrast to CT scan no preparation is required except for good head position. The head is cleaned with saline where the probe is applied if necessary. The TCS probe is applied directly on the skin where the craniotomy defect (in the temporal region) is and images in coronal plane are obtained. To visualize the brain images, as described by Seidel et al. [1] and by Couture et al. [2]: we positioned the ultrasound probe over the temporal area in an axial orientation with the marker pointing toward the occiput. Then using 2D imaging, we adjusted the display depth to see the opposite skull border. Following these initial steps, we scanned in a cranial to caudal direction to identify the intracranial bone artifacts: contralateral skull, foramen lacerum, petrous ridge, and sphenoid bone. Then we tilted at about 10° cranially to identify the 3rd ventricle. Color Doppler using a low-velocity scale is then used to visualize the main cerebral arteries along the Circle of Willis. Nine days after surgery, weaning of the EVD was attempted. Prior to EVD closure, TCS showed a measurement of the 3rd ventricle at around 1.16 cm (Fig. 1b). On the 3rd day of weaning, she deteriorated clinically and the TCS showed a dilated 3rd ventricle measuring 1.37 cm (Fig. 1c). This correlated well with the CT scan (Fig. 1d) and with clinical signs of active hydrocephalus as both her sensorium and communication were affected. Subsequently following EVD re-opening, on the next day using TCS the 3rd ventricle dimension was reduced to 0.99 cm (Fig. 1e) and the following day it went down to 0.69 cm (Fig. 1f). After 20 days from the hemorrhage, the EVD was clamped and removed because her sensorium and ICP were well preserved during the EVD weaning process. Patient had a favorable outcome and was transferred to a rehabilitation center.

## Discussion

Hydrocephalus is a common problem after subarachnoid hemorrhage SAH [3]. It is a devastating complication and can occur in up to 30% of patients. Its onset can be acute, within 48 h of SAH or, less frequently chronic, developing weeks or even months after SAH. The pathophysiology is most likely due to acute or chronic obstruction of cerebrospinal fluid (CSF) flow either by clot burden or induced inflammation and arachnoiditis. There are no current guidelines on how to manage or follow these patients in the acute stage and how to prevent the development of chronic symptomatic hydrocephalus. CT scan is used frequently in the management and monitoring of acute hydrocephalus of SAH as these patients often comatosed or sedated and clinical examination is not the safest method to monitor for hydrocephalus. According to the Food and Drug Administration, there is no amount of radiation that is completely without risk of radiation related cancer [4]. Moreover, transfer from and to intensive care units pose a real problem to medical care staff. Therefore, alternative monitoring strategies should be developed. The role of TCS has evolved as an imaging modality for the brain parenchyma in the setting of multiple pathologies, including movement disorders, parkinsonism and follow up of vasospasm post SAH using transcranial Doppler [5]. The diagnosis and monitoring for hydrocephalus has been used in young children because of the possibility of using the fontanelle as an acoustic window to the brain. The advantages of TCS in the management of hydrocephalus in adult have not been fully studied to date. But according to sporadic papers, the advantages of using TCS even in adults to follow hydrocephalus could encompass ease of use, low cost, wide acceptance by patients, no radiation risk, high mobility and relative independence from movement artifacts. Specifically, bedside availability and reliability has made critical care intensivists interested to use it with increasing frequency [6].

The accuracy of ultrasound compared to CT scan is not well established in adults and no known large studies confirming or infirming the utility of ultrasound in adults with hydrocephalus. Most of the literature comparing ultrasonography and computed tomography has been done in infants and children because of the natural acoustic windows of the brain through the fontanelles [7–12]. A report in 1984 by Rubenstein describes 19 adult patients the correlation between ultrasound images of the brain obtained post-operatively through a burr-hole and computed tomography [13]. The quality of the image was, however, much inferior to what we are able to see now. Recently, a comparison of the two techniques was performed in 15 brain-injured patients [14]. In 15 patients with brain injury, the 3rd ventricle was 35.5 ± 12 mm using TCS versus 33.1 ± 14 mm with computed tomography with an intraclass correlation of 0.88 and 95% confidence interval of 0.63–0.96, *p* < 0.01. There were no systematic biais using the Bland–Altman plot. In our case, the craniectomy site allowed direct skin dura matter contact which helps clarify more the brain structures and decreases the distance and eliminates artifacts caused by bone. To our knowledge, so far not many case reports and or case series evoked the possibility to use ultrasound instead of CT scan to monitor these patients’ hydrocephalus. Recently, a case series was published and showed that TCS using B-sonography mode was effective in decision making regarding removal of external ventricular drainage in patients with post-hemorrhagic malabsorption hydrocephalus with good specificity and sensitivity [15, 16].

This case illustrates the usefulness of hand-held bedside ultrasound for follow-up of patients with subarachnoid hemorrhage as a non-invasive, simple and safe method to monitor for the development of hydrocephalus and may be to guide management.

## Conclusion

Patients with SAH and particular those with a craniotomy can be monitored easily at the bedside with hand-held TCS for the development of hydrocephalus. Further studies with larger number of patients should be performed to evaluate the feasibility, sensitivity and specificity of TCS in the detection of hydrocephalus in adult patients after SAH.

## Abbreviations

CSF: cerebrospinal fluid
CT: computed tomography
EVD: external ventricular drain
ICP: intracranial pressure
SAH: subarachnoid hemorrhage
TCS: transcranial sonography
